# 

**Published:** 1888-11

**Authors:** 


Whole Number, CCCXXVIII.

**November, 1888.**

No. 4. VOL. XXVIII. New Series.

LIBRARY INDEXED S.G.O.

### Buffalo Medical and Surgical Journal.

Established 1845 by AUSTIN FLINT, M. D.

*Editors:*

THOS. LOTHROP, M. D. W. W. POTTER, M. D.

*Associate Editors:*

FLOYD S. CREGO, M. D. EDWARD B. ANGELL, M. D., Rochester, N.Y.

SCRIPTA FERUNT ANNOS

PUBLISHED MONTHLY.

BUFFALO:

1888.

Entered at the Post-Office at Buffalo, N. Y., as Second-class Mail Matter.

*Times Printing House, Buffalo, N. Y.*

